# Bouveret syndrome: A challenging case of impacted gallstone within the fourth part of the duodenum

**DOI:** 10.1016/j.ijscr.2022.107084

**Published:** 2022-04-14

**Authors:** Emma Smith, Sarah Zhao, Michael El Boghdady, Serena Sabato-Ceraldi

**Affiliations:** a General Surgery Department, Croydon University Hospital, London CR7 7YE, United Kingdom; b University of Edinburgh, Scotland, United Kingdom

**Keywords:** Bouveret syndrome, Gallstone ileus, Enterolithotomy, Emergency general surgery, Case report

## Abstract

**Introduction and importance:**

Bouveret syndrome is a rare condition characterised by gastric outlet obstruction secondary to a gallstone fistulating into the proximal duodenum or pylorus. Although rare, this condition carries a high mortality rate and no current standardised guidelines for management.

**Case presentation:**

We present a case of a patient in their 60s with recurrent small bowel obstruction secondary to a cholecysto-duodenal fistula and large gallstone which became impacted in the fourth part of the duodenum. The patient had a P-POSSUM Score of 14% mortality and 60% morbidity risk, had multiple co-morbidities, was bedbound, BMI 59 and had been deemed high risk for general anaesthetic at oncology centre for a 10 × 10 cm likely gynaecological malignancy a month prior to this admission.

**Clinical discussion:**

In contrast to existing literature, endoscopic lithotripsy was considered but not attempted due to unavailability of this service locally. Surgical intervention was decided based on radiological features of impending duodenal perforation on CT imaging and multiple disciplinary team discussion. The patient was managed with open enterolithotomy at the duodeno-jejunal (DJ) flexure and discharged 3 weeks post-operatively at her pre-operative baseline.

**Conclusion:**

This is the first report to our knowledge to describe successful surgical management of a gallstone impacted in the fourth part of the duodenum. In cases where anatomical location of impaction precludes retrieval via simple gastrostomy, we suggest using high pressure flush to mobilise the stone to more favourable location distally. We emphasise that stone size should be considered when planning surgical management.

## Highlights

• Consider Bouveret syndrome as a differential in patients with small bowel obstruction, and pneumobilia even without history of gallstones.
• Imaging often underestimates the full size of ectopic gallstones and thus risk of impaction may not be fully evaluated.
• Management requires multidisciplinary team involvement between radiology, surgical, endoscopist and anaesthetic specialties.
• Enterolithotomy can be considered for large, impacted stones and can be a safe option in patients with multiple morbidities.
• We suggest techniques using high pressure flush to mobilise inaccessible stones to a more favourable distal location.

## 1 Introduction

Bouveret syndrome is a rare cause of gallstone ileus, accounting for 2–3% of cases [1]. It is characterised by fistulation of a gallstone into the proximal duodenum with resultant gastric outlet obstruction [2]. Common presenting features are nausea, vomiting, and epigastric pain. Other signs include anorexia and haematemesis [3]. Radiologically, patients may have Rigler's triad of bowel obstruction, pneumobilia and ectopic gallstone on CT or plain radiograph [4]. There is slight female preponderance and average age is 68.8 years [5]. Notably, not all patients will have prior history of cholelithiasis - this is typically present in only 48–63% of cases [6].

Although rare, this syndrome carries a high mortality rate of 12–33% [1]. This may be due to a combination of patient co-morbidities and technically challenging surgery. In this report, we present a case of Bouveret syndrome requiring an atypical approach to surgical retrieval due to distal stone location. This case has been reported in line with the SCARE criteria [7].

## 2 Presentation of case

A female patient in her 60s was brought in by ambulance with abdominal pain and vomiting. She had a previous admission 6 days prior with gallstone ileus confirmed on non-contrast CT abdomen and pelvis imaging (patient had reduced renal function). A cholecysto-duodenal fistula at the second part of the duodenum (D2) and further gallbladder calculi were described. The previous episode was managed conservatively with spontaneous passage of a 2 × 1 cm gallstone into the colon after two days of observation.

Past medical history included hypertension, lymphoedema, arthritis, gout, and suspected left-sided ovarian malignancy (deemed high risk for cancer resection under general anaesthetic from Multi-Disciplinary Team discussion at a major oncology centre). Regular medications included amlodipine and omeprazole. Her BMI was 59, she was bed-bound with home package of care (due to limb lymphoedema), she was a non-smoker and consumed minimal alcohol.

On examination, she had localised right upper quadrant abdominal guarding, multiple episodes bilious vomiting and was clinically stable. Laboratory work up showed white cell count (WCC) of 17.4 × 10^9^/l, neutrophil count 13.0 × 10^9^/l and C-Reactive Protein (CRP) 174 mg/dl. Lipase level, liver function tests, lactate and urea and electrolytes levels were within normal range.

**Fig. 1:**
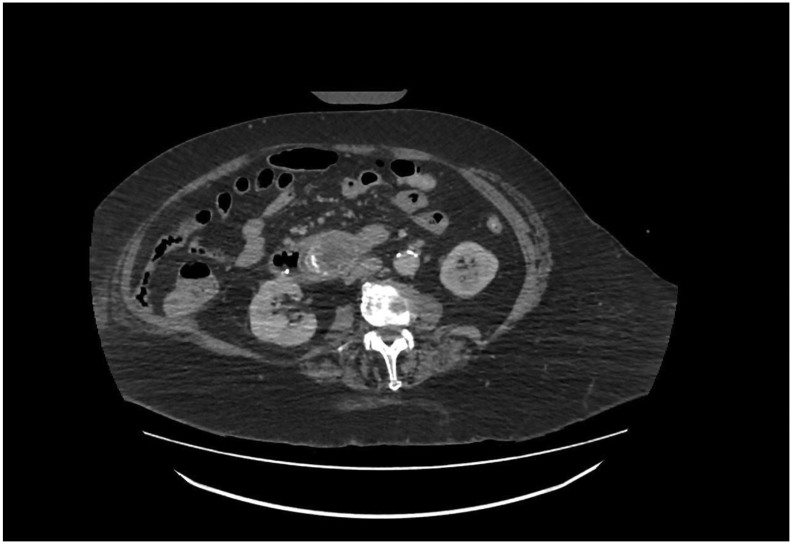
CT abdomen pelvis on admission. Axial slice of CT abdomen pelvis on admission demonstrating large, impacted gallstone within distal duodenum.

**Fig. 2:**
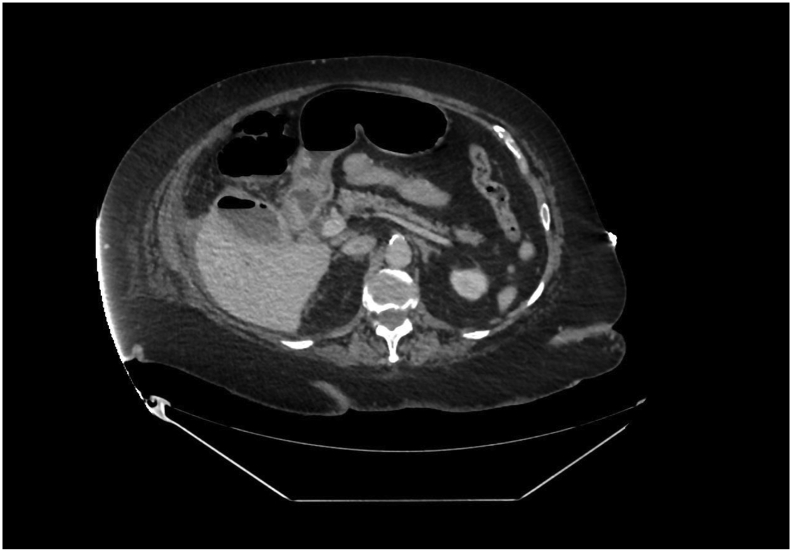
CT abdomen pelvis on admission. Axial slice of CT abdomen pelvis on admission demonstrating air fluid level within gallbladder.

**Fig. 3:**
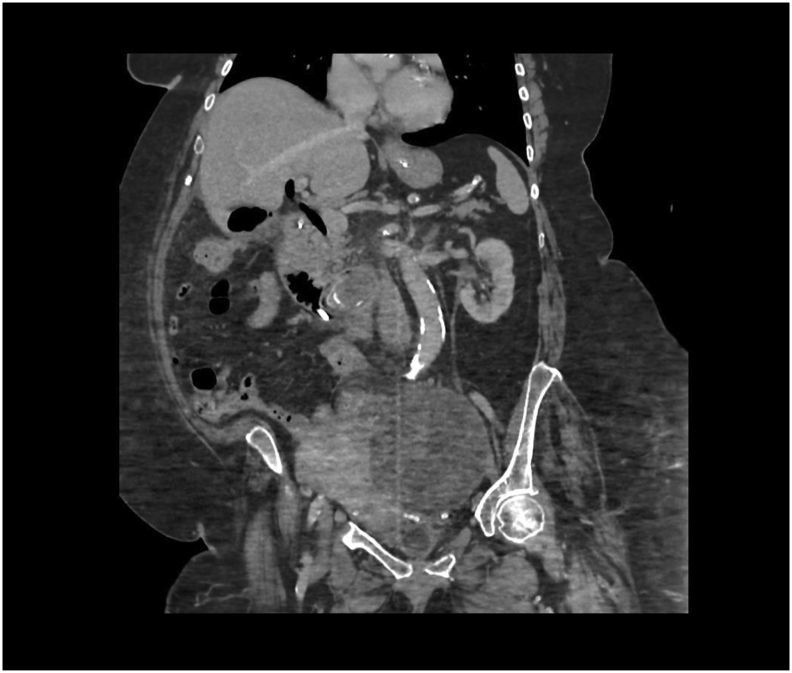
CT abdomen pelvis on admission. Coronal slice of CT abdomen pelvis with visible ectopic gallstone and cholechoduodenal fistula.

Intravenous contrast-enhanced CT abdomen and pelvis performed during readmission showed a persistent 3 cm gallstone in D2 with cholecysto-duodenal fistula, thickened gallbladder consistent with cholecystitis, sigmoid diverticulitis, and further irregularity of the ovarian mass margins. There was no evidence of perforation. Interval CT imaging 5 days later re-estimated the gallstone size at 5 cm with migration into the fourth part of duodenum (D4) (Fig. 1, Fig. 2, Fig. 3).

Following resuscitation, the patient was managed with nasogastric drainage, oral intake cessation, total parenteral nutrition, and antibiotics. This was due to high anaesthetic risk (P-POSSUM Score = 14% mortality, 60% morbidity) and concern of prolonged ventilatory wean in intensive care. On day 5 of admission, decision was made to operate given clinical deterioration, radiological signs of pressure necrosis and impending duodenal perforation (highlighted by consultant gastrointestinal radiologist). Although endoscopy is a primary management option in Bouveret syndrome, particularly in high-risk patients, an acute endoscopic service was not available locally and attempted transfer to a tertiary centre would have led to unacceptable delay in definitive treatment. Surgical technique was discussed pre-operatively with regional hepato-pancreato-biliary (HPB) centre and decision was made not to attempt concurrent cholecystectomy. The remaining gallstones were deemed small and anticipated not to cause further obstruction. Also, morbidity risk would increase with longer operating time, considering the added technical difficulty arising from the cholecysto-duodenal fistula. The gynae-oncology team advised that adnexal mass biopsy was inappropriate due to seeding risk and low influence on long-term management.

**Fig. 4:**
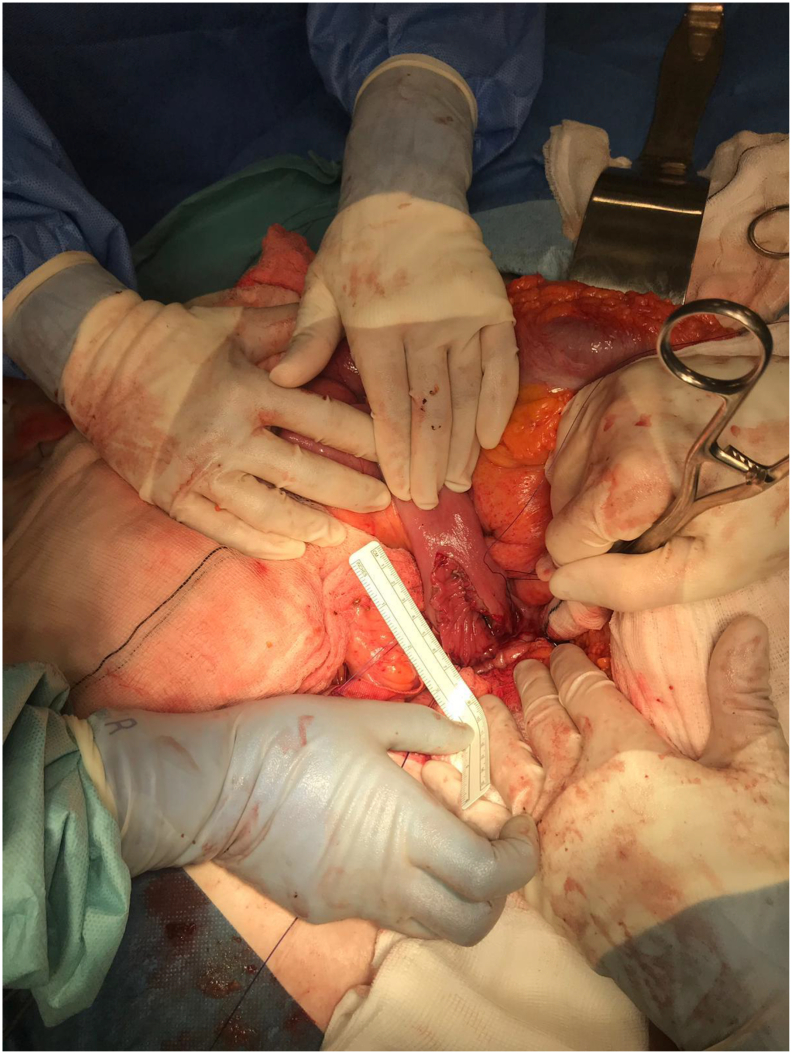
Intra-operative surgical site photograph. Photograph of open jejunotomy site for stone extraction during emergency laparotomy.

**Fig. 5:**
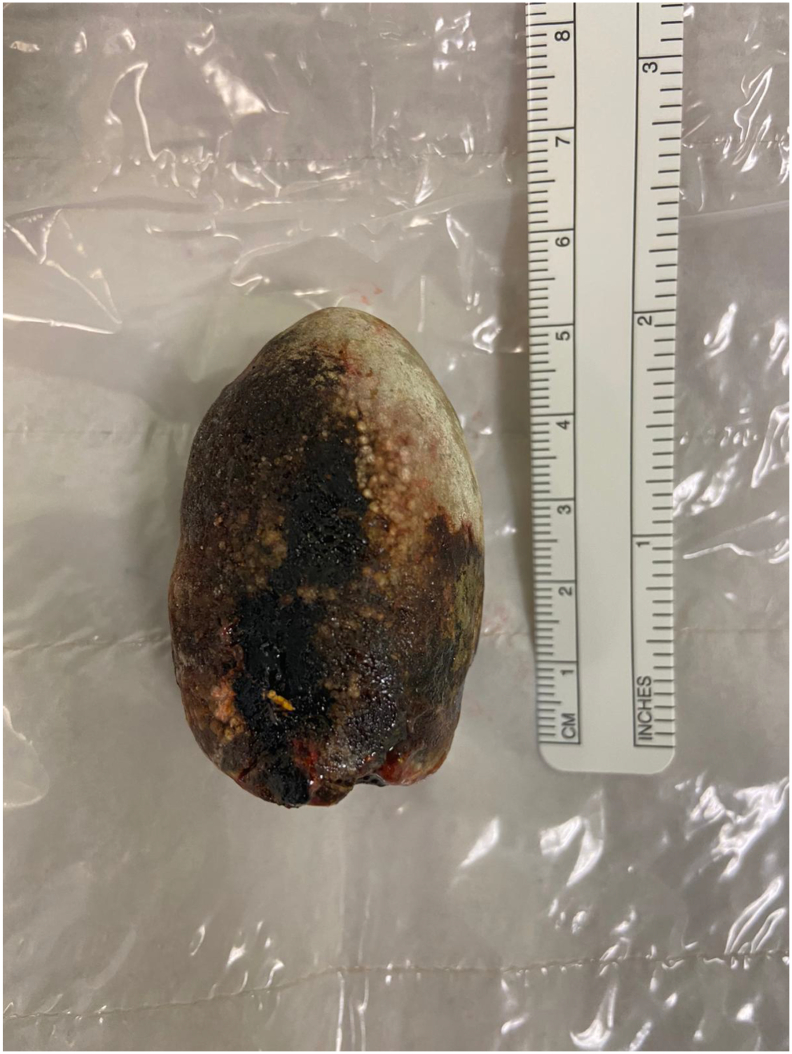
Specimen photograph. Photograph of retrieved 5.5 cm ectopic gallstone specimen against ruler.

Emergency laparotomy was performed by consultant upper gastrointestinal surgeon on day 6 of admission via extended upper midline incision. Duodenal kocherization and visualisation of the gallbladder were not possible due to severe adhesions, cholecysto-duodenal fistula, and risk of injury to surrounding structures. Unsuccessful attempt was made to dislodge and milk the stone proximally for retrieval via pyloroplasty, as recommended by HPB centre. Subsequently, high-pressure 50 ml water flushes were delivered via gastrostomy to expel the stone distally past the duodenojejunal flexure. It was then retrieved here via jejunotomy (Fig. 4). The stone was pigmented, calcified and 5.5 × 4 cm (Fig. 5). The gastrostomy was closed using Heineke-Mikulicz pyloroplasty and the jejunotomy was closed with continuous 4/0 PDS suture. Water leak test was negative. Two post-operative Robinson's drains were placed near both enterotomy sites. Total estimated intraoperative blood loss was 400 ml.

The patient required 2-day admission to the high dependency unit and resumed soft diet at 12 days post-operatively. Minor post-operative complications included stage 1 acute kidney injury and 4 × 2 cm abdominal wall haematoma both managed conservatively (Clavien-Dindo Classification Grade I [8]). Repeat CT imaging on post-operative day 8 showed no intra-abdominal collection or leak. She was discharged 3 weeks post-operatively at her functional baseline and remained clinically well when followed up at four-month post-operatively.

5 months after discharge she was readmitted under acute medical team with obstructive uropathy secondary to progression of ovarian malignancy (requiring bilateral nephrostomies). She deteriorated and was ultimately discharged to a palliative hospice. Patient passed away 5 months after initial surgical discharge from end-stage ovarian malignancy. Cause of death was unrelated to surgical post-operative complications.

## 3 Discussion

Other reported cases of Bouveret syndrome similarly describe elderly patients with multiple comorbidities. The most common site is cholecystoduodenal (60%), then cholecystocolic (17%), cholecystogastric (5%) and choledochoduodenal (5%) [9], [10]. Considering the low incidence and variance in anatomy and presentation, there are no current standardised management guidelines. Surgery carries high complication risk due to the demographic, diagnostic delay, and challenging technique. Additionally, concurrent pathologies such as diverticulitis and cholecystitis (as in this case), can exacerbate diagnostic pitfalls [4], [11].

If tolerated, oral contrast or magnetic resonance cholangiopancreatography imaging can help delineate local biliary tree anatomy [1], [12]. As in this case, it is important to recognise that the true gallstone size is often underestimated radiologically if only the calcified parts are visualised [6], leading to underestimation of the risk of mechanical obstruction. According to the literature, stones of ≥2.5 cm carry higher risk of impaction and further risk of obstruction with reduced intestinal luminal diameter (i.e. duodenum) [13]. Thus, recognising in advance the significance of stone size in relation to impending obstruction is important for employing timely (and potentially subsequently less-invasive) management. With our case, the timing of intervention was influenced by several factors including: availability of endoscopic services, local availability of higher level post-operative care and patient co-morbidities (including pre-existing nutritional deficit and prolonged starvation period). The latter was addressed in the pre-operative optimisation, with commencement of total parenteral nutrition via peripheral line. The scenario was further complicated by COVID-19 pandemic restraints enforced during that time.

The consensus is that endoscopic treatment should be attempted where possible. This includes basket stone removal and various lithotripsy modalities (mechanical, electrohydraulic and extracorporeal shockwave) [10]. Endoscopy can also be an adjunct to surgery to aid stone mobilisation to a more favourable extraction site. Although minimally invasive, these approaches have logistical limitations and require an experienced endoscopist. Only two thirds of stones can be visualised endoscopically, with even fewer amenable to endoscopic extraction. Lithotripsy fragments can also migrate and cause further obstruction distally [2], therefore any remaining bowel should be examined intraoperatively. Indeed, it has been reported that endoscopic success rates are low and 91% will require further surgical management [14]. Endoscopic retrieval was not feasible in our case due to the large and distal gallstone.

When clinically suitable (not in our case), laparoscopy can help identify the stone before converting to open procedure for enterolithotomy. Retrieval via gastrostomy is first line if the stone can be milked proximally into the stomach [2], otherwise distal extraction is required via enterotomy. If duodenotomy performed, then Heineke-Mikulicz closure technique can reduce stenosis risk [15]. Both these techniques were required in this case due to the challenging gallstone location. It was difficult to safely access the retroperitoneal D4 level for manual manipulation and thus high-pressure intraluminal flushes were used to mobilise it distally. Occasionally, larger defects are required and in these cases Roux-en-Y reconstructions and Jaboulay gastroduodenostomy have been performed [8].

There is debate as to whether cholecystectomy (with fistula excision) should be attempted during primary surgery or performed as interval procedure [1], [2], [4], [16]. Recurrence of gallstone ileus prior to second stage definitive surgery is reported around 5% [4] and consideration should be given to size and number of remaining calculi. However, recurrence rates in simple enterolithotomy without fistula repair are reported as <5%, with only 10% requiring re-operation for persistent symptoms [17]. The latter was favoured in our case considering the significant local inflammation, low likelihood of complications from the persisting fistula (remaining gallstones ≤5 mm), and high peri-operative risk. Overall, management decisions should be made via multidisciplinary approach.

## 4 Conclusion

In our case, there was satisfactory surgical outcome with the described technique for a distally located stone in D4 despite invasive management, recurrent disease, and concurrent pathologies. The risk of perforation and nutritional complications secondary to prolonged obstruction must be balanced with intervention risk. This case also highlights the importance of recognising radiological underestimation of stone size within this risk assessment and planning surgical intervention accordingly.

## Consent

Written informed consent was obtained from the deceased patient's next of kin for publication of this case report and accompanying images. A copy of the written consent is available for review by the Editor-in-Chief of this journal on request.

## Provenance and peer review

Not commissioned, externally peer-reviewed.

## Ethical approval

N/A.

## Funding

None.

## Guarantor

Ms Serena Sabato-Ceraldi, serena.ceraldi@nhs.net.

## Research registration number

N/A.

## CRediT authorship contribution statement

Emma Smith: Methodology, Investigation, Data curation, Writing – Original draft
Sarah Zhao: Investigation, Data curation, Writing – Original draft, Visualization
Michael El Boghdady: Writing – Review & editing, Project administration, Visualization
Serena Sabato-Ceraldi: Writing – Review & editing, Supervision.

## Declaration of competing interest

The authors confirm there are no conflicts of interest associated with this report.
